# Cancer treatment-induced NAD+ depletion in premature senescence and late cardiovascular complications

**DOI:** 10.20517/jca.2022.13

**Published:** 2022-04-29

**Authors:** Priyanka Banerjee, Elizabeth A. Olmsted-Davis, Anita Deswal, Minh TH. Nguyen, Efstratios Koutroumpakis, Nicholas L. Palaskas, Steven H. Lin, Sivareddy Kotla, Cielito Reyes-Gibby, Sai-Ching J. Yeung, Syed Wamique Yusuf, Momoko Yoshimoto, Michihiro Kobayashi, Bing Yu, Keri Schadler, Joerg Herrmann, John P. Cooke, Abhishek Jain, Eduardo Chini, Nhat-Tu Le, Jun-Ichi Abe

**Affiliations:** 1 Academic Institute, Department of Cardiovascular Sciences, Center for Cardiovascular Sciences, Houston Methodist Research Institute, Weill Cornell Medical College, Houston, TX 77030, USA.; 2 Department of Cardiology, The University of Texas MD Anderson Cancer Center, Houston, TX 77030, USA.; 3 University of Science and Technology of Hanoi, Vietnam Academy of Science and Technology, Hanoi 122100, Vietnam.; 4 Department of Radiation Oncology, The University of Texas MD Anderson Cancer Center, Houston, TX 77030, USA.; 5 Department of Emergency Medicine, The University of Texas MD Anderson Cancer Center, Houston, TX 77030, USA.; 6 Center for Stem Cell & Regenerative Medicine, The University of Texas Health Science Center of Houston, TX 77030, USA.; 7 Department of Epidemiology, Human Genetics and Environmental Sciences School of Public Health, The University of Texas Health Science Center of Houston, TX 77030, USA.; 8 Department of Pediatrics, The University of Texas MD Anderson Cancer Center, Houston, TX 77030, USA.; 9 Cardio Oncology Clinic, Division of Preventive Cardiology, Department of Cardiovascular Medicine, Mayo Clinic, Rochester, MN 55905, USA.; 10 Department of Biomedical Engineering, Texas A&M, College Station, TX 77843, USA.; 11 Department of Anesthesiology and Perioperative Medicine, Mayo Clinic, Jacksonville, FL 32224, USA.

**Keywords:** NAD^+^, senescence-associated secretory phenotype (SASP), cardiovascular diseases, p90RSK, ERK5

## Abstract

Numerous studies have revealed the critical role of premature senescence induced by various cancer treatment modalities in the pathogenesis of aging-related diseases. Senescence-associated secretory phenotype (SASP) can be induced by telomere dysfunction. Telomeric DNA damage response induced by some cancer treatments can persist for months, possibly accounting for long-term sequelae of cancer treatments. Telomeric DNA damage-induced mitochondrial dysfunction and increased reactive oxygen species production are hallmarks of premature senescence. Recently, we reported that the nucleus-mitochondria positive feedback loop formed by p90 ribosomal S6 kinase (p90RSK) and phosphorylation of S496 on ERK5 (a unique member of the mitogen-activated protein kinase family that is not only a kinase but also a transcriptional co-activator) were vital signaling events that played crucial roles in linking mitochondrial dysfunction, nuclear telomere dysfunction, persistent SASP induction, and atherosclerosis. In this review, we will discuss the role of NAD^+^ depletion in instigating SASP and its downstream signaling and regulatory mechanisms that lead to the premature onset of atherosclerotic cardiovascular diseases in cancer survivors.

## INTRODUCTION

Late cardiovascular complications such as myocardial infarction and structural heart defects were observed in childhood cancer survivors treated at St. Jude Children’s Research Hospital. The cumulative cardiovascular disease (CVD) incidence of those who survived more than 10 years and reached age over 18 years was 45.5% relative to 15.7% of the age- and sex-matched controls. These findings revealed the increased CVD risks in cancer survivors relative to their age and sex-matched controls. This study also revealed the association between high cardiac radiation exposure (≥ 35 Gy) and high CVD risks^[1,2]^.

Growing evidence demonstrates that various cancer treatment modalities, including chemotherapy, radiation therapy, targeted therapy, immunotherapy, hormone therapy, stem cell or bone marrow transplant, and surgery^[3–5]^, cause premature senescence, as reviewed elsewhere^[6–8]^.

A few examples are listed here. Radiation therapy triggers premature senescence rather than apoptosis in human non-small cell lung cancer (NSCLC) cells through the activation of p53-p21 signaling. A small molecule inhibitor, Nutilin-3, mediates p53 activation and sensitizes NSCLC cells to radiation therapy, leading to ionizing radiation (IR)-mediated premature senescence^[9]^. A CDK4/6-specific inhibitor palbociclib (PD033299)^[10]^ induces premature senescence in various cancer cell types by phosphorylating and activating the transcription factor Forkhead Box M1 (FOXM1)^[10–13]^. A second-generation selective Aurora kinase inhibitor, Alisertib (MLN8237)^[14]^, induces premature senescence in cells lacking p53 and p73^[15–17]^. Further, immunotherapy mediates cancer cell growth arrest via IFN-γ- and TNF-induced premature senescence^[18–20]^.

From the perspective of the Fibonacci mathematical modeling, hand grip strength (HGS) can be a physical biomarker or an indicator of aging^[2,21]^. A meta-analysis of 53,476 participants^[22]^ revealed that HGS was associated with reduced all-cause mortality. This association appeared to be compromised in participants with an average age of 60. As such, cancer treatment-induced premature senescence can be reflected by HGS and the association with increased incidence of CVD, all-cause mortality, and cardiovascular mortality. An impaired physical function, as evidenced by the decreased 6-minute walking distance (6MWD), was observed in long-term survivors after allogeneic hematopoietic stem cell transplantation (allo-HSCT) relative to their siblings^[23]^. In the peripheral blood of cancer survivors, an increased level of both serum interleukin 6 (IL-6) and CDKN2A [p16(INK4A)] was noted^[24–26]^. These observations suggest the association between allo-HSCT and premature senescence^[23–26]^.

Diverse cancer treatment modalities are utilized in different cancer types^[3–5]^. However, survivors of different cancer treatment modalities exhibit common phenotypes with late cardiovascular complications^[27–31]^. Among cancer patients, who had or had not been previously exposed to known cardiotoxic agents, (i) those who survived after chemotherapy agents without acute cardiotoxicity (as defined by Children’s Oncology Group guidelines) also exhibited increased cardiac dysfunction, body mass index, fasting serum non-high-density lipoprotein cholesterol, insulin, and C-reactive protein compared to non-cancer siblings; (ii) those who survived after chemotherapy agents with acute cardiotoxicity exhibited similar phenotypes as described in (i). These data demonstrated that chemotherapy agents with acute cardiotoxicity only contributed little to CVD incidence including hypertension, dyslipidemia, and obesity^[32]^. Without pre-exposure to acute cardiotoxicity-inducing agents, cancer survivors also exhibited late cardiovascular complications. These findings suggested that, by inducing premature senescence, different cancer treatment modalities can cause common cardiovascular complications long after the completion of cancer treatments^[33–35]^.

## WHAT IS SENESCENCE?

For the first time in 1961, Hayflick and Moorhead introduced the original concept of senescence based on their observation that the proliferation of human diploid fibroblasts was irreversibly arrested after serial passage *in vitro*. This type of time-dependent growth or proliferation arrest was termed replicative senescence (RS)^[36,37]^. Further studies revealed that both internal and external stimuli, including cellular stress, reactive oxygen species (ROS), radiation, and mitochondrial dysfunction, can induce cell cycle arrest, i.e., “stress-induced premature senescence (SIPS)”^[38,39]^. RS and SIPS may follow different molecular mechanisms and time frames. RS is accompanied by the shortening of telomeres until a critical length at which senescence is induced, known as the Hayflick limit^[40]^. SIPS may or may not be associated with telomere shortening^[41–44]^. For example, the telomerase-immortalized human foreskin fibroblast (hTERT-BJ1) cells exposed to ultraviolet B light or H_2_O_2_ develop SIPS, suggesting that SIPS can be independent of telomerase activity and telomere shortening^[42]^.

It is important to emphasize that both telomeric and non-telomeric DNA damage contribute to the induction of cellular senescence with time; both senescence and organismal aging are accompanied by increased DNA damage, as evidenced by γH2AX foci formation^[45]^. Nakamura *et al.*^[45]^ examined the chromosomal location of senescence-associated γH2AX-foci that may be found at either uncapped telomeres or non-telomeric DNA damage in human and murine cells, and found that telomeric and non-telomeric DNA damage responses (DDR) play equivalent roles in inducing senescence, which is mainly regulated by telomere length rather than species differences. It is also important to note that genomic DNA damage is repaired relatively faster (within 24 hours) than telomeric DNA damage^[46,47]^. Accordingly, telomeric DNA damage can persist for longer periods of time and can cause a persistent DDR for months^[48]^. As such, the long-lasting effect of cancer therapy-induced SISP may be explained by this delayed and sustained telomeric DDR.

## TELOMERE SHORTENING AND DYSFUNCTION IN CANCER SURVIVORS

Length of telomeres, the TTAGGG repeats at eukaryotic chromosome ends, shortens with age until reaching the Hayflick limit^[49,50]^. Telomere shortening is associated with cancer treatments and age-related diseases^[51–54]^ and has been a marker of cellular senescence marker^[6,49]^. The St. Jude Lifetime Cohort Study (SJLIFE) revealed a significantly shorter telomere length in leukocytes of childhood cancer survivors relative to that of the age- and sex-matched controls^[54]^. Patients with solid malignancies received immune checkpoint inhibitors revealed a link between peripheral leukocyte telomere shortening and poor survival^[55]^. Mammalian telomeres are protected by Shelterin, a protein complex composed of six proteins including TERF telomeric repeat binding factor 1 (TRF1)^[56]^, TERF telomeric repeat binding factor 2 (TRF2)^[57]^, telomeric repeat-binding factor 2-interacting protein 1 [TERF2IP, or repressor/activator protein 1 (RAP1)]^[58]^, the protection of telomeres 1 (POT1)^[59]^, TPP1^[60]^, and TRF1-interacting nuclear factor 2 (TIN2)^[61,62]^. Structure and biological function of these six Shelterin proteins have been extensively reviewed elsewhere^[61–68]^. Briefly, TRF1 and TRF2 recognize and bind the TTAGGG repeats via interacting with the TRF homology (TRFH). With specific docking sites, the TRFH domains facilitate TIN2 and TERF2IP binding to TRF1 and TRF2. POT1 binds the telomere 3′-overhang and TPP1 forming a quadruplex structure. POT1 serves as the inhibitor of telomeres^[61–68]^.

DDR recognizes DNA damage to activate pathways to repair the damage. Double-strand breaks (DSB) can be repaired by (i) non-homologous end joining (NHEJ) machinery that joins two chromosomal ends with no or minimal base-pairing at the junction; and/or by (ii) 5′-to-3′ resection of the DSB ends to generate 3′-ended single-stranded DNA tails, which are then repaired by homology-dependent recombination pathways^[64–68]^. Through binding telomeric DNA at the chromosome ends, Shelterin protects telomeric DNA from being recognized as DNA damage by DDR. Loss of Shelterin protective effects and/or telomere shortening leads to telomere dysfunction. Dysfunctional telomeres are recognized by DDR and repaired by NHEJ machinery and/or homology-dependent recombination apparatus. As a result, chromosomal abnormalities are generated, cell cycle arrest is induced, p53 signaling is upregulated, and the PPARγ co-activator 1-α and -β (PGC1- α and β) is repressed. Consequently, mitochondrial biogenesis and function are hampered, leading to the upregulation of mitochondria ROS production. Therefore, mitochondrial dysfunction and elevated levels of ROS can be early events in premature senescence induced by telomere dysfunction^[69]^.

Different from proliferative cells, post-mitotic cardiomyocytes develop senescent-like phenotypes through a mechanism independent of cell division and telomere length. As characterized by persistent telomeric DNA damage, post-mitotic cardiomyocyte senescence can be mediated by mitochondrial dysfunction, which activates p21^CIP^ and p16^INK4a^, resulting in a non-canonical Senescence-associated secretory phenotype (SASP)^[70,71]^. Studies revealed the critical role of cardiomyocyte mitochondria in cardiac function^[72]^. As radiation therapy induces cardiomyocyte mitochondrial dysfunction^[73,74]^, it is reasonable to speculate that cancer treatments induce post-mitotic cardiomyocyte senescence through persistent telomeric DNA damage-mediated mitochondrial dysfunction, independent of cell division and telomere length.

## CANCER TREATMENTS INDUCE PREMATURE SENESCENCE IN VARIOUS CELL TYPES

Numerous *in vitro* and *in vivo* studies have shown that cancer treatments including chemotherapy and radiation therapy can induce premature senescence in different cell types^[75]^. For instance, cancer cell senescence was detected in clinical cancer samples of breast cancer patients after preoperative neoadjuvant chemotherapy. Cyclin-dependent kinases 4/6 (CDK4/6) small molecule inhibitors mediated the induction of cancer cell senescence^[8]^. IR induces endothelial cell senescence, as evidenced by decreased NO production and thrombomodulin expression, increased adhesion molecule expression, elevated ROS production and inflammatory cytokines, and impairment of proliferative capacity as well as the formation of capillary-like structure. Endothelial cell senescence can cause endothelial dysfunction through dysregulation of vasodilation and hemostasis, inducing oxidative stress and inflammation and inhibition of angiogenesis, which are involved in IR-mediated late effects^[76]^. Doxorubicin and IR induce myeloid cell senescence by triggering metabolite changes with nicotinamide adenine dinucleotide (NAD) depletion and mitochondrial stunning^[77]^. Low doses of doxorubicin induce human primary vascular smooth muscle cells (VSMC) senescence through the mediation of TRF2 ubiquitination and proteasomal degradation^[78]^. Doxorubicin and IR induce stem cell premature senescence by mediating expression of the senescence marker p16(INK4a) in human cardiac progenitor cells, and consequently impair their regenerative capacity, leading to cardiotoxicity and heart failure in cancer survivors^[79]^. Cancer treatments induce SIPS not only in cardiovascular cells, but also in stem cells, which may have an important role in the long-lasting effects of cancer treatments leading to CVD.

## STEM CELL PREMATURE SENESCENCE AND EXHAUSTION IN CANCER SURVIVORS

Stem cells are cells with self-renewal properties for an unlimited or prolonged period of time and have the potential to differentiate into other cell lineages^[80]^. Stem cell exhaustion and diminished activity of hematopoietic stem cells (HSCs) are hallmarks of aging^[81,82]^. Thus, the overall disease-free survival after bone marrow transplantation depends on the age of stem cell donors, i.e., young donors can provide recipients with longer disease-free survival^[83,84]^. Importantly, hematopoietic cell transplantation (HCT) causes significant stress on HSC^[6]^, leading to SIPS, and subsequently reduces HSC’s potency to repopulate^[85,86]^. HCT also elicits telomere shortening in HSCs of the recipients, irrespective of myeloablative or non-myeloablative conditioning regimens^[85]^.

## UNIQUE FEATURES OF SENESCENCE-ASSOCIATED SECRETORY PHENOTYPE

Senescent cells communicate with neighboring cells, such as immune and cancer cells, through secreting cytokines, chemokines, matrix metalloproteinases *etc.*, as well as through a direct intercellular protein transfer (IPT)^[87]^. Particularly, senescent cells secrete a cocktail of proinflammatory cytokines, chemokines, growth factors, pro-angiogenic factors, ROS, and proteases *etc.*, namely senescence-associated secretory phenotype (SASP). These secreted cytokines and chemokines recruit T cells, macrophages, and natural killer cells, which help remove senescent cells. Of note, the timely clearance of senescent cells is critical in tissue homeostasis, in which immune cells play a vital role^[88–90]^. In a direct IPT process, proteins from senescent cells are directly transferred to neighboring cells, activating signaling pathways in neighboring cells, ultimately changing neighboring cell behaviors^[87]^.

Unlike apoptotic or quiescent cells, senescent cells exhibit high metabolic activity. Studies demonstrated that high metabolic activity directs energy toward activities related to senescent state, including the induction of SASP and the modulation of immune responses within the senescent microenvironment. Like cancer cells, the glycolytic state in senescent cells, for example, in senescent human diploid fibroblasts (HDF), was higher than in their young counterparts, even in high oxygen conditions^[91,92]^. Senescent human HDF displayed an increased expression of key glycolytic enzymes including hexokinase, phosphoglycerate kinase, and phosphoglycerate mutase^[93–95]^.

Metabolic activity is controlled at various levels. The oxidized state of NAD (NAD^+^) is a vital cofactor that controls metabolic activities using its electron transfer function in redox reactions. Functioning as a co-enzyme, NAD^+^ regulates glycolysis, tricarboxylic acid (TCA, Kreb’s) cycle, and fatty acid oxidation to form NAD^+^ hydrogen (NADH)^[96,97]^. In glycolysis, NAD^+^ is reduced to form NADH^+^H^+[96]^. Numerous studies showed that cellular NAD^+^ levels are reduced in senescent cells, inducing premature senescence and age-related diseases^[98,99]^. Our recent study revealed that in myeloid cells, NAD^+^ is reduced after treatment with doxorubicin or IR. Specifically, we observed a sustained SASP induction and an upregulation of p90RSK-mediated ERK5 S496 phosphorylation as well as downstream inflammatory signaling pathways in myeloid cells treated with doxorubicin or IR^[77]^. To gain insight into the underlying molecular mechanism, we discovered that doxorubicin and IR activated poly (ADP-ribose) polymerase (PARP) and subsequent NAD^+^ depletion. Consequently, reversible mitochondrial dysfunction was mediated without inducing cell death even when ATP is depleted. We also noted that, although low-dose IR inhibited both oxidative phosphorylation (OXPHOS) and glycolysis without causing cellular necrosis or apoptosis, significant upregulation of mitochondrial ROS production and succinate production occurred, attesting to the metabolically active status of SASP^[77]^.

## THE BALANCE BETWEEN NAD^+^ BIOSYNTHESIS AND CONSUMPTION MAINTAINS THE NAD^+^ LEVEL

### NAD^+^ biosynthesis

**Figure 1.**
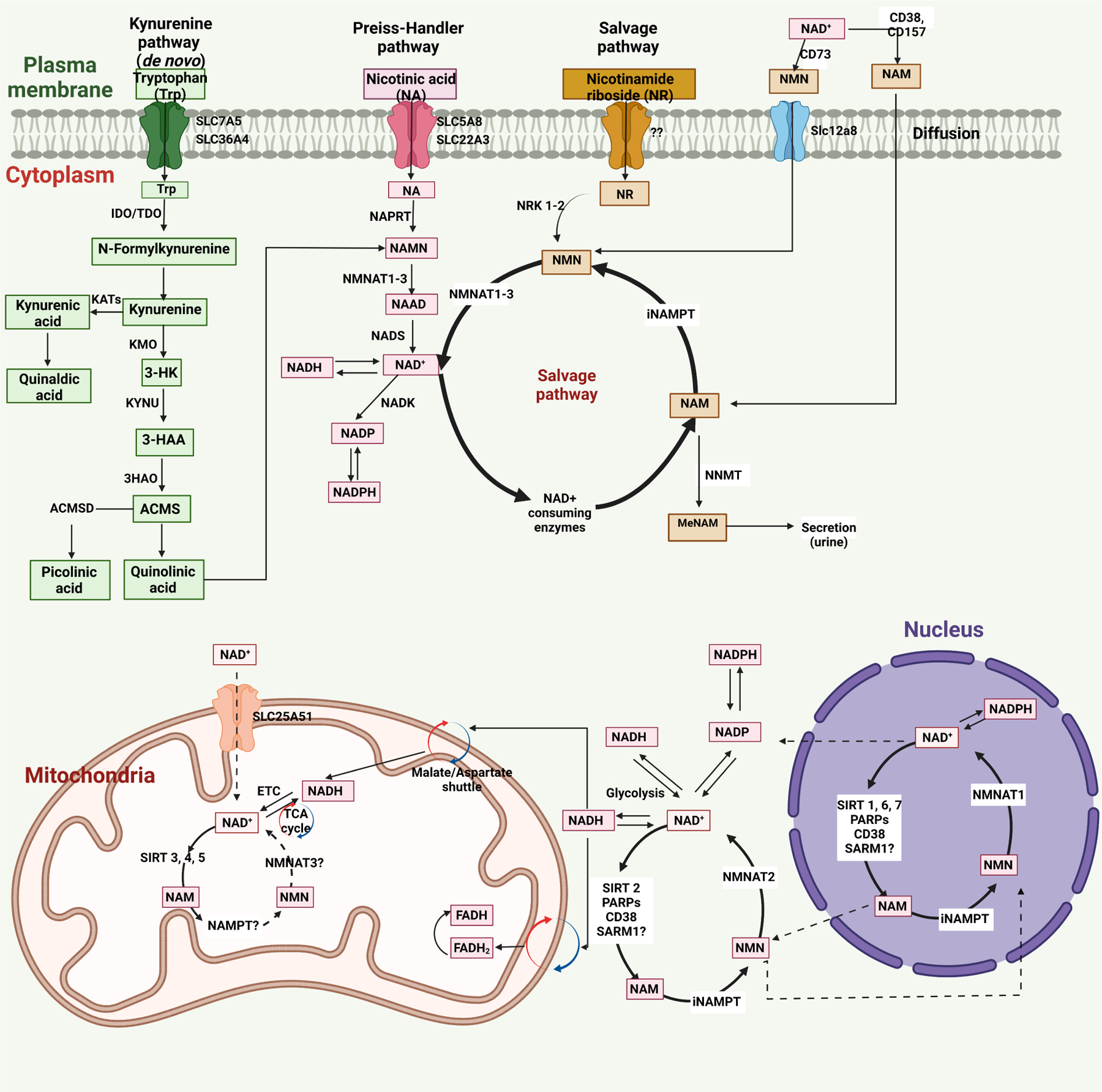
NAD^+^ metabolism in cells and its compartmentalization^[99,103]^. Human cells produce NAD^+^ through three major pathways: the Kynurenine pathway, Preiss-Handler pathway, and Salvage pathway. In the Kynurenine pathway, which is a de novo pathway, the precursor molecule, tryptophan (Trp), after entering the cells via the transporters SLC7A5 and SLC36A4, is converted to N-formyl kynurenine (FK) by the rate-limiting enzyme indoleamine 2,3- dioxygenase (IDO) or the rate-limiting enzyme tryptophan 2,3- dioxygenase (TDO) and then FK is converted to kynurenine. The kynurenine aminotransferases (KATs) convert kynurenine to kynurenic acid, further converted to quinaldic acid. In addition to this, kynurenine 3-monooxygenase (KMO) converts kynurenine to 3- hydroxykynurenine (3-HK), which is further transformed to 3- hydroxy anthranilic acid (3-HAA) by tryptophan 2,3- dioxygenase (KYNU). The 3-HAA gives rise to α- amino- β- carboxy muconate ε- semialdehyde (ACMS) by the enzyme 3-hydroxyanthranilic acid oxygenase (3HAO). Finally, ACMS is transformed to picolinic acid by α-amino-β-carboxy muconate-ε-semialdehyde decarboxylase (ACMSD) or quinolinic acid. In the Preiss-Handler pathway, the precursor molecule nicotinic acid (NA) first enters the cells via SLC5A8 or SLC22A3 transporters. It is then converted to nicotinic acid mononucleotide (NAMN) by the enzyme nicotinic acid phosphoribosyltransferase (NAPRT), which is then converted into nicotinic acid adenine dinucleotide (NAAD) by the enzymes called nicotinamide mononucleotide adenylyl transferases (NMNAT1, NMNAT2, and NMNAT3). Next, NAD^+^ synthase (NADS) transforms NAAD to NAD^+^. The NAD^+^ can be directly phosphorylated by NAD^+^ kinase (NADK) to produce NADP(H). In the Salvage pathway, the intracellular nicotinamide (NAM) is recycled back to NAD^+^ via the formation of nicotinamide mononucleotide (NMN) by intracellular nicotinamide phosphoribosyltransferase (iNAMPT). The NAM is the byproduct generated by the NAD^+^ consuming enzymes, sirtuins, poly (ADP-ribose) polymerases (PARPs), CD38, CD157, and SARM1. The Salvage pathway also uses nicotinamide riboside (NR) to produce the NMN via the enzyme nicotinamide riboside kinases 1 and 2 (NRK1 and NRK2). The cellular NAD^+^ level is balanced by biosynthesis and consumption in different subcellular compartments. For example, in the cytoplasm, the intracellular NAMPT (iNAMPT) converts NAM to NMN, further transformed to NAD^+^ by another cytoplasm-specific enzyme, NMNAT2. The NADH, generated from the NAD^+^ in the cytoplasm and utilized by Glycolysis, is transported to the mitochondria via the malate/aspartate shuttle. Via the electron transport chain (ETC), the NADH is oxidized to NAD^+^ by mitochondria specific complex I, while by tricarboxylic acid (TCA) cycle, the NAD^+^ is transformed to NADH. The mitochondrial SIRT 3, 4, 5 convert NAD^+^ to NAM. The NADH can enter the mitochondria via the glyceraldehyde 3- phosphate shuttle and results in reduced flavin adenine dinucleotide (FADH2), which is converted to the FADH mitochondrial complex II. The mitochondrial transporter SLC25A51 can also help the direct mitochondrial entry of NAD ^+^. The nuclear NAD^+^ pool equilibrates with the cytosolic NAD^+^ pool by diffusion through the unidentified nuclear pore^[99,103]^. The nuclear enzymes SIRT 1, 6, 7, and PARPs, CD38, SARM1, consume NAD^+^ and regulate the NAD^+^ homeostasis in the nucleus.

There are three independent biosynthetic pathways for generating NAD^+^, namely, *de novo* biosynthesis from tryptophan, Preiss-Handler pathway, and NAD^+^ salvage pathway^[100]^. The *de novo* (kynurenine) pathway uses tryptophan, which enters the cell via plasma membrane transporters SLC7A5 and SLC36A4 [Figure 1]. The functional contribution of kynurenine pathway to the production of NAD^+^ remains unclear, because not all the enzymes related to kynurenine pathway are expressed in most cells besides liver and immune cells including macrophages. Nicotinamide (NAM) generated by tryptophan metabolism in the liver is released into the circulation and is taken up by the other cells for conversion to NAD^+^ via the salvage pathway as described below.

The second pathway to generate NAD^+^ is Preiss-Handler pathway and the dietary nicotinic acid (NA), which enters the cell via SLC5A8 or SLC22A13 transporters, and is used as the precursor to produce NAD^+^ by the Preiss–Handler pathway and converted to nicotinic acid mononucleotide (NAMN) by NA phosphoribosyl-transferase (NAPRT). NAMN is the common intermediate produced by kynurenine and Preiss-Handler pathway and is converted to the nicotinic acid adenine dinucleotide (NAAD) by nicotinamide mononucleotide adenylyl transferases (NMNAT1, NMNAT2 and NMNAT3). Finally, NAD^+^ synthetase (NADS) converts NAAD into NAD^+^ [Figure 1].

The third pathway is the salvage pathway, which generates NAD^+^ not only from extracellular nicotinamide riboside (NR) or nicotinamide mononucleotide (NMN) but also recycles NAM to NMN, which is metabolized to produce NAD^+^ by nicotinic acid mononucleotide transferases (NAMNTs). In the extracellular space, the ectoenzymes CD38 and CD157 convert NAD^+^ to NAM, and then NMN [by extracellular nicotinamide phosphoribosyltransferase (eNAMPT)]. CD73 dephosphorylates NMN, generates nicotinamide riboside (NR), imports into the cells by an unknown transporter, then forms NMN via nicotinamide riboside kinases 1 and 2 (NRK1 and NRK2) In addition, there is an NMN-specific transporter (SLC12A8)for importing NMN into the cell. After all, NMNAT1–3 converts NMN to NAD^+^ [Figure 1].

### Subcellular compartment-specific NAD^+^ metabolism

There are subcellular compartment-specific NAD^+^-consuming or -generating enzymes, and subcellular NAD^+^ homeostasis and level are regulated by subcellular compartmentalization. For example, intracellular NAMPT (iNAMPT) and NMNAT2 localize in the cytoplasm and generate NAD^+^ in the cytoplasm. NMNAT isoform of NMNAT3 specifically localizes in the mitochondria. Also, NAD^+^-dependent mitochondrial Sirtuin 3 (SIRT3), SIRT4 and SIRT5 can consume NAD^+^ and covert it to NAM in the mitochondria. There is a nucleus-specific NMNAT isoform (NMNAT1) that converts NMN to NAD^+^.

Although there is subcellular location-specific regulation of NAD^+^ by subcellular-specific enzymes, NAD^+^ levels in each subcellular compartment are also co-regulated by various shuttling mechanisms amongst compartments. Recent studies showed that the mammalian NAD^+^ mitochondrial transporter SLC25A51 plays a crucial role in intact NAD^+^ uptake from the cytoplasm to mitochondria^[101]^. The malate/aspartate shuttle system can also shuttle NAD/NADH between cytoplasm and mitochondria. The cytosolic NADH imported to mitochondria via the malate/aspartate shuttle is oxidized by complex I in the electron transport chain (ETC) and converts back to NAD^+[99,102,103]^. The nuclear NAD^+^ pool equilibrates with the cytosolic NAD^+^ pool by diffusion through the nuclear pore, but the details of this mechanism remain unclear^[99,103]^.

### NAD^+^-consuming enzymes and aging process

NAD^+^ concentration decreases during aging in humans and in animal models^[104–107]^. An increased NAD^+^ availability by NAD^+^ precursors counteracts the effects of aging in various experimental models^[108,109]^, demonstrating the critical role of NAD^+^ depletion in aging. NAMPT expression declines with aging, supporting the involvement of NAMPT in NAD^+^ depletion^[110–112]^. As NAMPT plays an important role in regulating circadian oscillation, the decline of circadian oscillation with aging may be indirectly involved in aging-mediated NAD^+^ depletion^[113]^. Recent studies suggested that NAD^+^-consuming enzymes may also play a regulatory role in aging-mediated NAD^+^ depletion. These NAD^+^-consuming enzymes are sirtuins, poly (ADP-ribose) polymerases (PARPs), Sterile Alpha and TIR Motif Containing 1 (SARM1), and the ectoenzymes CD38 and CD157^[114,115]^.

#### Sirtuins

Sirtuins (SIRTs, or SIR2), NAD^+^-dependent deacetylase or mono-ADP-ribosyltransferase, catalyze the NAD^+^-dependent deacetylation of lysine on the target protein^[116]^. Mammalian SIRTs consist of 7 members (SIRT1–7) with distinct subcellular localization, enzymatic activity, and downstream targets^[99,117]^. In the nucleus, SIRT1, 6, 7 consume NAD^+^ to make NAM. Subsequently, NAM is converted into NMN by iNAMPT, which is used to produce NAD^+^ by NMNAT1^[99]^. In mitochondria, SIRT3, 4, 5 consume NAD^+^ to make NAM; however, it is unclear whether NAM is converted into NMN and NAD^+99]^. SIRT1 downregulates SASP factors such as IL6 and IL8 by increasing acetylation of Histone H3 (K9) and H4 (K16)^[118]^. With aging, the SIRT level is decreased^[119]^. In endothelial cells (EC), SIRT1, 6 inhibition induces premature senescence^[120,121]^. SIRT activity can be increased using pharmaceutically active compounds (SIRT-activating compounds, or STACs) such as resveratrol, SRT1720, SRT3025, and SRT2104. These compounds may be useful in the management of cardiomyopathy, atherosclerosis, metabolic syndrome, and endothelial dysfunction^[122]^.

#### Poly (ADP-ribose) polymerases (PARPs)

PARPs are expressed in all eukaryotes except yeast and are involved in numerous cellular processes such as DNA repair and apoptosis, gene regulation, and chromatin remodeling. PARPs can transfer one (mono) or more (poly) ADP-ribose moieties from NAD^+^ to substrates to form poly (ADP-ribose) (PAR) chains with varying lengths and contents. There are 17 PARP isoforms that share a conserved catalytic domain with various domains such as zinc finger, BRCT, SAM, SAP, ankyrin and macro domain^[123–125]^. PARPs consume NAD^+^ and convert NAD^+^ to NAM^[99,124]^.

PARP1 is the best characterized PARP member. PARP1 activation plays distinct roles based on the context. In a normal (non-stressed) condition, PARP1 protects the replication fork. In aging, PARP1 maintains telomere length and telomerase activity^[126,127]^. As a sensor of DNA damage, PARP1 activation increases in response to DNA damage^[123,128]^ and mediates NAD^+^ depletion^[99]^. PARP1 activation is involved in various DDR mechanisms such as single-strand break (SSB) repair, DSB repair, homologous recombination (HR), and NHEJ. In a stressed (DNA damage) condition, activated PARP1 recruits DDR machineries such as scaffold protein XRCC1 (in case of SSB), and MRE11, EXO1, BRCA1, and BRCA2 (in case of DSB)^[129]^. Through activating PARP1 and the downstream NF-κB signaling, anti-melanoma DNA-damaging drugs induce melanoma cell SASP^[130]^. However, excessive DNA damage leads to PARP1 overactivation, severe NAD^+^ depletion, and cell death. PARP1-dependent DNA damage-induced programmed cell death pathway, namely “parthanatos”, has been implicated in heart diseases^[131]^. In cancer cells, such as breast and ovarian cancers, HR is less effective due to mutations in DNA repair genes, e.g., BRCA1, BRCA2. Consequently, DNA damage accumulates and induces genome instability. These cancer cells utilize other DDR systems including PARP1. In such cases, PARP1 inhibitors will be helpful as they cause cellular apoptosis and eliminate the mutant, damaged cells^[132]^. As such, PARP inhibitors, e.g., isoindolinone-based PARP inhibitor INO-1001 (ClinicalTrials.gov. NCT00271765, NCT00271167), and Olaparib (NCT03782818) are in early stages of evaluation for the treatment of atherosclerotic CVD and pulmonary arterial hypertension.

#### CD38 and CD157

Both CD38 and CD 157 are paralogues, and both are located on chromosome 4 (4p15)^[133]^. The CD38 type II transmembrane protein is an ectoenzyme^[134]^, which has multiple functional roles in tumorigenesis and aging by regulating NAD^+^ levels and extracellular nucleotide homeostasis^[134]^. This ectoenzyme is on the cell surface with its catalytic site facing towards the extracellular environment. CD38 can also regulate the NAD^+^ metabolism by regulating the metabolism of its extracellular precursor, such as nicotinamide mononucleotide (NMN)^[105]^. CD38 mRNA and protein expression along with CD38 NADase enzymatic activity are significantly increased during the process of aging. Furthermore, CD38 induction correlates with NAD^+^ depletion in liver, adipose tissue, spleen, and skeletal muscles in aged mice^[105]^.

CD157 is expressed on myeloid cells, B-cell progenitors, and endothelial cells^[99]^. *In vitro* macrophage polarization from M0 to proinflammatory M1 macrophages exhibited increased expression of CD38 and to a smaller extent CD157 with lesser NADase activity. Like CD38, CD157 has also been suggested to consume extracellular NAD^+[133]^. However, it is becoming clear that CD157 consumes mostly the NAD^+^ precursor nicotinamide riboside (NR)^[135]^ and is not a significant NAD^+^ consumer^[105,115,136,137]^.

Both CD38 and CD157 expression are increased in the epididymal white adipose tissue from aged (25-month-old) wild-type mice as compared to 6-month-old mice^[137,138]^. Therefore, it is possible that the expression of CD38 and CD157 plays a significant role in aging-mediated NAD^+^ depletion. CD38 is activated in the endothelial cells in heart by hypoxia-reoxygenation and triggers NAD^+^ depletion^[139]^ and endothelial cell dysfunction. In *in vivo* models, the CD38 inhibitors (thiazoloquin(az)olin(on)es and luteolinidin) can block the CD38 activity and prevent endothelial and myocardial cell damage in the post-ischemic heart^[137,140–142]^.

#### Human sterile alpha and HEAT/Armadillo motif containing 1 (SARM1)

SARM1 has multiple functional domains including a mitochondrial targeting signal (MTS), an auto-inhibitory N-terminus region with armadillo motifs (ARM) and HEAT motifs, two sterile alpha motifs (SAM), and Toll/interleukin-1 receptor (TIR) domain. SARM1 has two different types of NADase enzymatic activities for (1) hydrolyzing NAD^+^ to NAM and ADP-ribose (ADPR), and (2) ADP-ribosyl cyclase activity and generating NAM and cyclic ADPR from NAD^+^ by utilizing the TIR domain^[143]^. The NADase activity of SARM1 is regulated by phosphorylation. NAM acts as a feedback inhibitor of SARM1 NADase activity^[96]^.

SARM1 is reported to be a key mediator of axonal degeneration via the breakdown of NAD^+^ after neural injury or disease^[144]^, and SARM1 regulates the neuronal intrinsic immune response to axonal injuries through activating JNK-c-Jun signaling^[145]^. In oxidative stress, activated JNK phosphorylates SARM1, thereby increasing its NADase activity, reducing NAD^+^ levels and suppressing mitochondrial respiration^[146]^. Recently, it has been reported that the depletion of SARM1 inhibited NMNAT2-deficiency mediated axonopathy during the process of aging without any phenotypic manifestations^[147]^. Sur *et al.*^[148]^ have reported an important role of SARM1-induced inflammatory response in age-dependent susceptibility to rotenone-induced neurotoxicity. These data suggest that SARM1 plays a crucial role in increased susceptibility to age-associated neuronal loss. In the context of cytotoxic chemotherapy for cancer, the induction of chemotherapy-induced peripheral neuropathy (CIPN) by axonal degeneration may be due to the loss of NAD^+^ via SARM1 activity^[149]^.

SARM1 is also expressed at high levels in neurons in the brain and is linked to neuronal cell death after deprivation of glucose, ischemia, viral infection, or axonal damage^[150–152]^. Declines in cellular NAD^+^ levels and the rate-limiting enzyme NAMPT in NAD^+^ biosynthesis may play pathogenic roles in age-related cognitive decline, and treatment with NMN to increase NAD^+^ may improve cognition in the setting of aging. Chemotherapy-induced cognitive impairment (CICI) has been reported in almost 3/4 of cancer patients treated with chemotherapy, and a significant fraction of patients have continued cognitive decline. The metabolic pathways involving NAD^+^ contribute significantly to CICI, and treatment with NMN to increase NAD^+^ may prevent CICI^[153]^.

## NAD^+^ DEPLETION AND AGING PROCESS

NAD^+^ levels decline during aging and this decline can be linked to aging-related diseases, including atherosclerosis, arthritis, diabetes, cognitive dysfunction, and cancer. The relationship between NAD^+^ and various hallmarks of aging has been extensively reviewed elsewhere^[154]^. In this review, we will focus on the role of NAD^+^ depletion in “inflammaging” and telomere dysfunction.

## INFLAMMAGING

The term “inflammaging” refers to the systemic low-grade chronic inflammation status in the absence of infection. Inflammaging represents a central biological process in aging^[155,156]^ as well as the strong link between chronic inflammation and systemic metabolism including NAD^+^ depletion. First, studies have shown a strong correlation between NAD^+^ depletion with activation of innate immunity. CD38 expression increases with M1 (pro-inflammatory) polarization in macrophages, leading to a significant increase of NAD^+^ consumption and subsequent NAD^+^ depletion^[136,138,157]^. NAD^+^ precursors NMN and NR can inhibit glycolytic shifts, which are observed in M1 macrophage polarization, and CD38-mediated NAD^+^ depletion can attenuate the inflammatory response^[158]^. Aging is associated with a sustained increase in ROS, which upregulates NLRP3 inflammasome activation^[138,159,160]^. ROS induced by proinflammatory cytokines can also induce DNA damage, thus activating PARPs and CD38 and leading to aging-related NAD^+^ depletion. Therefore, enhanced expression of proinflammatory cytokines or ROS drives a vicious cycle of inflammaging by a positive feedback loop with activation of major consumers of NAD^+^, such as CD38 and PARPs, induced by ROS-mediated DNA damage and accelerated physiological age-related decline^[99]^. In contrast, M2 type-like (anti-inflammatory) macrophage increased NAMPT expression and subsequently upregulated NAD^+^ production^[138]^. Therefore, it is possible that M2 type-like macrophages can inhibit the process of inflammaging. Further investigation will be necessary to clarify these issues.

The aging process has a profound impact on adaptive immunity, and age-related immune dysfunction that includes remodeling of lymphoid organs and impairment of adaptive immunity is referred to as immunosenescence. Studies reported decreased levels of naïve T and B cells, and increased levels of memory T cells including cytotoxic CD8^+^CD28^-^ populations during the process of aging. This cytotoxic CD8^+^CD28^-^ population is characterized by inhibition of SIRT1 and FOXO1 levels^[161]^, which can be reversed by the inhibition of CD38^[162]^. Another type of T cells is also increased by aging, i.e., exhausted T cells. These T cells have increased expression of inhibitory receptor molecules (PD1 and TIM3) and a decrease in proliferative capacity and effector functions^[163,164]^. PD1 inhibitor can restore the effector function of aged T cells^[165]^. Both adoptive CAR-T and anti-PD1 immune checkpoint blockade mouse models demonstrated that NAD^+^ supplementation enhanced the tumor-killing efficacy of T cells *in vivo*. NAD^+^ supplementation may promote tumor-killing by tumor-infiltrated T cells after anti-PD1 immune checkpoint inhibitor treatment or adoptive chimeric antigen receptor (CAR) T cells through rescuing defective TUB-mediated NAMPT transcription^[166]^. Interestingly, in cancers that show resistance to PD1 inhibitor, CD38 expression is upregulated in exhausted CD8 T cell populations^[167,168]^, but a critical role of CD38-mediated NAD^+^ depletion in resistance to PD1 inhibitor cancer therapy needs further investigation.

## PERSISTENT SASP INDUCTION BY THE p90RSK-ERK5 S496 PHOSPHORYLATION-MEDIATED POSITIVE FEEDBACK LOOP AFTER CHEMO-RADIATION

**Figure 2.**
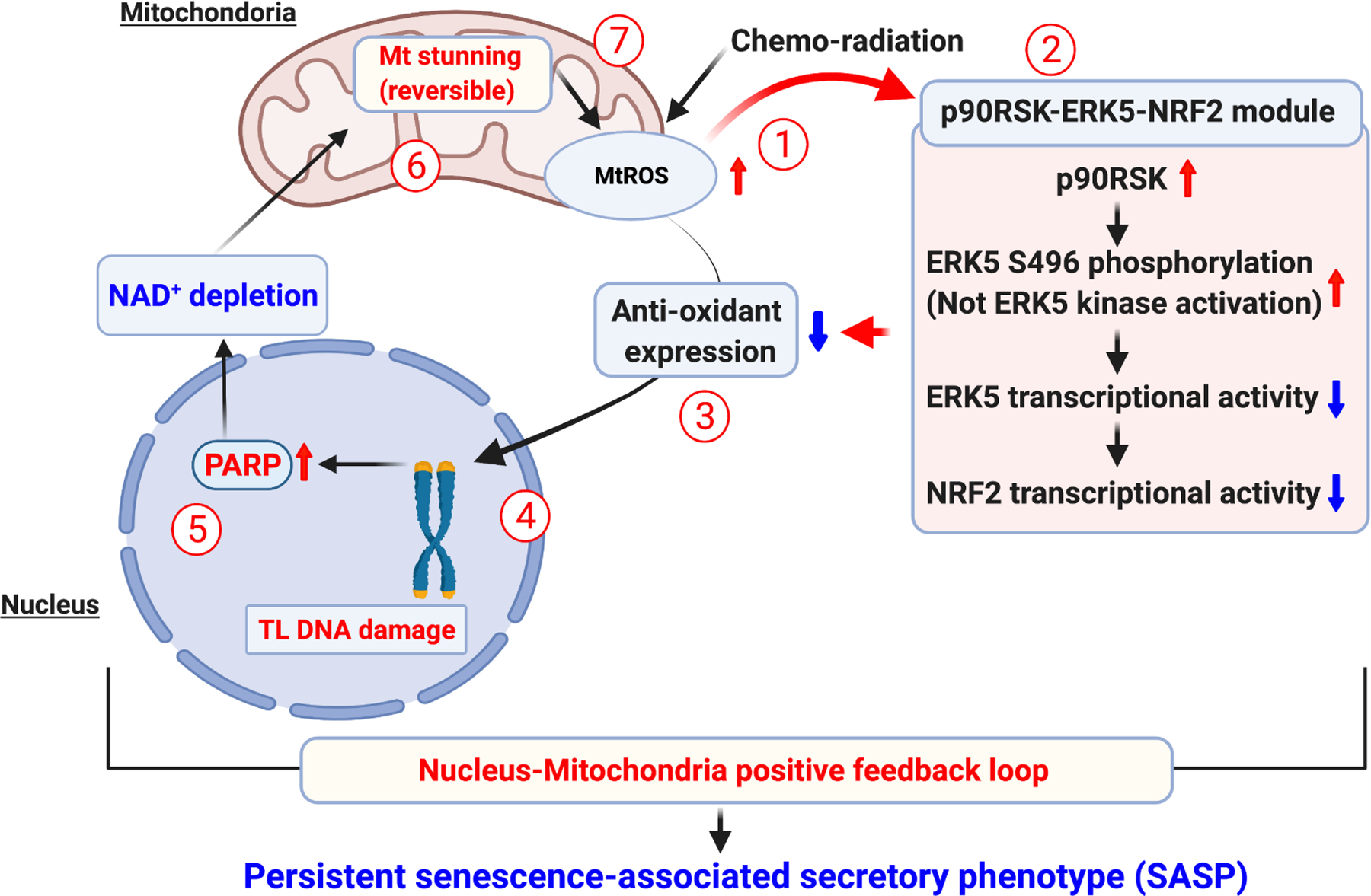
The SASP is sustained by a positive feedback loop constituted by p90RSK-ERK5 S496^[77]^. In the cells exposed to chemoradiation, a sustained SASP is maintained for the long term via the positive feedback loop. (1) The chemoradiation induces the production of mitochondrial ROS (mtROS) in the mitochondria, which in turn (2) increases the p90RSK phosphorylation leading to ERK5 S496 phosphorylation, the decreased transcriptional activity of ERK5, and reduced NRF2 transcriptional activity. Consequently, the (3) level of cellular antioxidant is dropped, causing (4) telomeric DNA damage, (5) PARP activation, and leading to NAD^+^ depletion. (6) The NAD^+^ depletion causes mitochondrial dysfunction, along with severe ATP depletion, termed reversible mitochondrial (mt) stunning, which further (7) causes mtROS production and reactivates the same p90RSK-ERK5-NRF2 module, thus constituting a positive feedback loop. This figure was modified from the figure in reference^[77]^

Telomere shortening and telomeric DNA damage induced by aging and cancer treatments can cause NAD^+^ depletion by activating PARP^[77]^ or increasing^[169]^ CD38 expression^[170]^. PARP1 overactivation can lead to a catastrophic decrease of cytosolic NAD^+^ and thereby directly inhibiting glycolysis and causing cell death^[117,171,172]^. Recently, however, we found that various cancer treatments (IR and doxorubicin) activate the p90RSK/ERK5-S496 inflammatory complex that leads to the formation of a positive feedback loop, which inducing mitochondrial stunning and a persistent SASP. This positive feedback loop is formed by the following steps (i) cancer treatments increase mitochondrial ROS production, (ii) mitochondrial ROS activates the p90RSK/ERK5-S496 complex and thereby decreasing NRF2 transcriptional activity; (iii) the reduction of NRF2 transcriptional activity inhibits antioxidant gene expression (HO1 and Trx1) that involve in the initiation of a persistent SASP including senescence, inflammation, mitochondrial ROS production, and impaired efferocytosis; (iv) steps (i) to (iii) is required for IR and doxorubicin to induce telomere shortening; (v) telomeric DNA damage activates PARP^[127]^; (vi) PARP activation causes mitochondrial damage and cell death^[148–150,173–175]^ [Figure 2]. As IR low dose and doxorubicin did not trigger immediate cell death, and the depletion of NAD^+^ and ATP was recovered by PARP and p90RSK inhibitors, this mitochondrial dysfunction is reversible. Accordingly, we referred this unique reversible form of mitochondrial dysfunction as “mitochondrial stunning”; (vii) cancer treatments-induced “mitochondrial stunning” was unique in that the cells remained metabolically active even in ATP-depleted conditions. Of note, an increased mitochondrial ROS and succinate production after IR low dose was sustained, and the late phase (but not early phase) of mitochondrial ROS and succinate production are p90RSK dependent. We also found that complex II activity is required for mitochondrial stunning-triggered mitochondrial ROS production. As such, sustained mitochondrial ROS production without killing the cells is critical for chronic inflammation and unceasing SASP status, which we noted even long after the completion of cancer therapy (late effects)^[77]^. A positive feedback loop formed by the p90RSK/ERK5-S496 inflammatory complex contributes to persistent SASP induction as (i) telomeric DNA damage to senescence; (ii) ERK5 S496 phosphorylation-NRF2 and PARP activation to inflammation and ROS^[176,177]^; (iii) mitochondrial stunning to mitochondrial ROS; and (iv) p90RSK-ERK5 S496 phosphorylation to efferocytosis [Figure 2]^[176]^. We anticipate that this positive feedback loop can explain the persistent SASP status seen in many cancer survivors with increased late CVD risks.

## AGING-RELATED DISEASES IN CANCER SURVIVORS

With the recent advancements in cancer detection and therapeutics, the life expectancy of patients with cancer has significantly increased^[178]^. Nearly 70% of patients with cancer will live at least 5 years from diagnosis and 18% will live 20 years or longer^[179]^. In 2016, almost 10 million elderly cancer survivors (≥ 65-year-old) were living in the US, while by 2040, the number is projected to grow to 19 million^[180,181]^. Even though this represents a great accomplishment of modern medicine, there is a cost to these accomplishments. Cancer and cancer treatments accelerate the process of aging in cancer survivors, manifesting as earlier onset and higher incidence of aging-related diseases including CVD, compared to the general population^[6]^.

Aging is a well-known risk factor for the development of coronary and peripheral artery disease, hypertension, heart failure, valvular disease, and atrial fibrillation^[182,183]^. The pathogenetic processes behind the above clinical manifestations include atherosclerosis, decreased arterial elasticity, arterial and myocardial fibrosis, calcification, and decreased myocardial relaxation^[183]^. These conditions are now seen significantly more often among cancer survivors, years after their cancer diagnosis and treatment. NAD^+^ biosynthetic and metabolic processes are mechanistically involved in aging, cancer, and many age-associated comorbidities^[184]^, and therapies aimed at raising intracellular NAD^+^ may remedy accelerated aging and age-associated diseases in cancer survivors. In fact, there are several ongoing clinical studies examining supplementation with NAD^+^ precursors as a therapeutic option for age-related diseases [Table 1].

Using reports on childhood cancer survivors makes it easier to disentangle the contribution of aging later in life from the direct effects of cancer and cancer therapies in the development of cardiovascular disease. In the Childhood Cancer Survivor Study, a retrospective study of over 10,000 adults who survived childhood cancer between 1970 and 1986, coronary artery disease was 10.4 times more frequent later in the life of cancer survivors compared to their siblings^[185]^. Furthermore, congestive heart failure was 15.1 and cerebrovascular accidents were 9.3 times more frequent in survivors of childhood cancer than their siblings^[185]^. A different study, using the registry of the Pediatric Oncology Group of Ontario Networked Information System, including over 7,000 childhood cancer survivors, showed that heart failure was 9.7 times more frequent in cancer survivors than in age-, gender-, and postal code-matched control individuals^[186]^. Additionally, coronary artery disease was 3.4, valvular disease 4.7 and arrhythmia 1.8 more frequent^[186]^. A smaller prospective study of 92 childhood cancer survivors from Germany further supports the theory of premature cardiovascular aging in cancer survivors. When compared to healthy controls, childhood cancer survivors had significantly reduced health-related physical fitness, significantly increased systolic and reduced diastolic blood pressure (wide pulse pressure) consistent with premature arterial stiffening, matching the cardiovascular phenotype of older individuals^[187]^. A different study including 19 long-term (> 10 years) high-risk neuroblastoma survivors revealed significantly higher levels of high-sensitivity CRP, which correlated with increased common carotid artery intima-media thickness in cancer survivors compared to age- and gender-matched controls, again another marker of premature cardiovascular aging^[188]^. Despite current evidence supporting higher prevalence and earlier onset of age-related CVD in cancer survivors, methodological challenges have limited the efforts to thoroughly study the aging-related consequences of cancer and cancer treatment^[189]^.

To overcome these challenges, in July 2018, the National Cancer Institute convened basic, clinical, and translational science experts who identified several research and resource needs that to be addressed immediately. The main items included: i) the need for longitudinal studies examining aging trajectories that include detailed data prior to, during, and post cancer treatment; ii) mechanistic studies that investigate the pathways leading to the development of aging phenotypes in cancer survivors; iii) long-term clinical surveillance studies to assess for late effects^[189]^. Addressing these needs will allow for a better understanding of aging in cancer survivors and will help to better identify, predict, and mitigate aging-related consequences of cancer and cancer treatment^[189]^. Yet there is a paucity of mechanistic investigations into the role of NAD^+^ metabolism and its regulatory pathways in accelerated aging of cancer survivors.

## MODELING CANCER TREATMENTS-INDUCED PREMATURE SENESCENCE WITH ORGAN-CHIPS

Organ-Chips are contemporary preclinical experimental models of disease and drug discovery. An organ chip is a microfluidic cell culture device typically consisting of two or more cell types arranged to simulate tissue- and organ-level physiology under continuous perfusion. These systems are capable of forming physiologically relevant tissue architecture in 3D and forming a tissue, thus recreating organ-level functionality not possible with conventional 2D or 3D culture systems. Importantly, they offer a reductionist approach to studying signaling pathways and are supported by high-resolution, real-time imaging and in vitro analysis of biochemical, genetic, and metabolic activities of living cells. The organ-on-chip technology of blood vessels has been transformational over the last decade; it has allowed functional analyses, continuous nutrition, intercellular transport, removal of byproducts, and secretion and biochemical assessment of co-cultured vascular cells not possible before with traditional experimental models^[190]^. Therefore, these systems are suited to model aging and senescence associated with cancer therapy in a manner complementary to animal models.

Recent works by Jain *et al.*^[193,195]^ demonstrated a 3D anatomical Vessel-Chip that supports the co-culture of EC and mural cells and recapitulates their bi-directional signaling under cyclic flow. The platform allows the construction of a wide range of luminal diameters and muscular layer thicknesses, thus providing a toolbox to create variable anatomy^[191–192,194]^. In this device, smooth muscle cells (SMCs) align circumferentially while ECs align axially under flow, as only observed *in vivo* in the past and in rare *in vitro* models. This system successfully characterizes the dynamics of cell size, density, growth, and alignment due to co-culture and shear. The matrix used in this system has bulk mechanical properties close to *in vivo* vessels. Another significant feature of our Vessel-Chip is that the subendothelial gap (distance between ECs and SMCs) of a Vessel-Chip is the same scale (~5µm-10µm in thickness) as *in vivo*, which is an important physiological feature. This platform technology can be used to include tissue-resident myeloid cells and other immune cells of the circulation and simulate senescence. The platform will also allow the investigation of changes in metabolites (e.g., NAD^+^ and its metabolites) in the microvascular microenvironment.

## CONCLUSION

Premature aging in cancer survivors is now well documented. In this current review, we discussed how various cancer treatments can modulate premature aging and induce SASP in these cancer survivors. Importantly, we also linked the role of NAD^+^ in the accelerated senescence and aging in these cancer patients. However, the role of NAD^+^ in different cell types and their cross talk contributing to the accelerated aging and SASP is yet to be explored. The outcome of several ongoing clinical trials on the drugs targeting the NAD^+^ and the related factors of that NAD^+^ metabolism pathway might provide some clue whether NAD^+^ can be a potential target for long-term management for inhibiting cardiovascular events in cancer survivors. Novel approaches, including the 3D anatomical Vessel-Chip model, can be a great tool for pre-assessment of the role of NAD^+^ depletion-mediated secreted factors in forming the interplay among different cell types, resulting in cancer therapy-induced vessel senescence.

## Figures and Tables

**Table 1.** Clinical trials with the NAD^+^ precursors in elderly people with or without diseases

Target molecule, therapy applied	Disease	Status/results	Reference
NAD^+^-precursors nicotinic acid, nicotinamide, and tryptophan	Aging	In physically compromised older adults, the dietary supplements did not alter the mitochondrial function or skeletal muscle function	NCT03310034 [196]
NAD3 (an over-the-counter dietary supplement	Aging	Yet to publish	NCT04276948
MIB-626 (Nicotinamide mononucleotide (NMN)	Alzheimer’s Disease, Dementia	Not yet recruiting	NCT05040321
Nicotinamide riboside (NR)	Aging	Yet to publish	NCT02950441
Niagen (Nicotinamide riboside, NR)	Mild Cognitive Impairment	Recruiting	NCT03482167
Deuterated nicotinamide (D4-NAM) IV infusion	To study the impact of lifestyle, aging on the rate of NAD synthesis	Recruiting	NCT04905446
Nicotinamide riboside (NR)	To study the role of dietary NR supplementation on the muscle functions in older people	Recruiting	NCT04691986
Nicotinamide riboside (NR)	Role of NR on the skeletal function and metabolic function in elderly people	Recruiting	NCT03818802
Nicotinamide riboside (NR)	Role of NR on the brain function, blood flow in elderly people with mild cognitive impairment	Yet to publish	NCT02942888
Nicotinamide riboside (NR)	Role of NR in metabolism and mitochondrial function in elderly people	Recruiting	NCT04907110
Nicotinamide riboside (NR)	To study whether NR supplementation can shorten the recovery time from acute injury	Recruiting	NCT04110028
Nicotinamide	Alzheimer’s Disease	Recruiting	NCT03061474
Nicotinamide	Alzheimer’s Disease	Yet to publish	NCT00580931
Niacin	Parkinsons Disease	Active, not recruiting	NCT03808961
Nicotinamide riboside (NR)	To study the role of NR on vascular function in young and older people following high-fat diet intake	Yet to publish	NCT03501433
Nicotinamide riboside (NR)	To assess the arterial stiffness in middle age and elderly people	Recruiting	NCT03821623
NMN	Aging	Yet to publish	NCT04228640
Nicotinamide riboside (NR)	Hypertension in elderly people	Recruiting	NCT04112043
Niagen	Aging, cognitive impairment	Active, not recruiting	NCT04078178
Niagen	Aging, age-associated physiological dysfunction	Yet to publish	NCT02921659
